# Artificial Intelligence in the Longevity Economy: What Do We Know?

**DOI:** 10.1093/geroni/igaf122.1367

**Published:** 2025-12-31

**Authors:** Taylor Brennan, Taylor Brennan

## Abstract

Artificial intelligence (AI) is transforming our society. As AI in the U.S. increasingly intersects with the growing longevity economy, it will continue influence public opinion and disrupt how consumers across the lifespan access information, work, care, and age (Coughlin, 2017). Aligned with GSA’s 2025 theme “Innovative Horizons in Gerontology,” this symposium presents findings from four MIT AgeLab studies exploring Americans’ attitudes towards AI in key gerontological domains. Using mixed methods and unique samples, the studies examine AI’s impact on older workers, family caregivers, aging in the latest phase of life, and information-seeking behavior at different ages. The first presentation highlights AI’s potential to support older workers, drawing on national interviews with HR professionals and company executives. The second presentation reports on a survey conducted with family caregivers exploring their use of and trust in AI for family caregiving. The third discusses how adults aged 85+ perceive and use AI for managing aging in the latest phase of life. The final presentation shares insights from a national public opinion survey on attitudes toward AI for information and advice, emphasizing age-related differences. Together, these presentations will deepen our understanding of how Americans engage with AI across diverse contexts of aging. A facilitated discussion will follow, encouraging attendees to reflect on key themes and emerging issues at the intersection of AI and the longevity economy.

